# Podophyllotoxin: History, Recent Advances and Future Prospects

**DOI:** 10.3390/biom11040603

**Published:** 2021-04-19

**Authors:** Zinnia Shah, Umar Farooq Gohar, Iffat Jamshed, Aamir Mushtaq, Hamid Mukhtar, Muhammad Zia-UI-Haq, Sebastian Ionut Toma, Rosana Manea, Marius Moga, Bianca Popovici

**Affiliations:** 1 Institute of Industrial Biotechnology (IIB), Government College University, Lahore 54000, Pakistan; syedazinniashah@gmail.com (Z.S.); dr.mufgohar@gcu.edu.pk (U.F.G.); buttiffat804@gmail.com (I.J.); hamidmukhtar@gcu.edu.pk (H.M.); 2 Gulab Devi Institute of Pharmacy, Gulab Devi Educational Complex, Lahore 54000, Pakistan; aamir_mushtaq@hotmail.com; 3 Office of Research, Innovation & Commercialization, Lahore College for Women University, Lahore 54000, Pakistan; 4 Faculty of Medicine, Transilvania University of Brasov, 500036 Brasov, Romania; moga.og@gmail.com (M.M.); biancadr@yahoo.com (B.P.)

**Keywords:** novel biomolecules, antitumor phytochemical, podophyllotoxins, chemotherapy, COVID-19, cytokine storm

## Abstract

Podophyllotoxin, along with its various derivatives and congeners are widely recognized as broad-spectrum pharmacologically active compounds. Etoposide, for instance, is the frontline chemotherapeutic drug used against various cancers due to its superior anticancer activity. It has recently been redeveloped for the purpose of treating cytokine storm in COVID-19 patients. Podophyllotoxin and its naturally occurring congeners have low bioavailability and almost all these initially discovered compounds cause systemic toxicity and development of drug resistance. Moreover, the production of synthetic derivatives that could suffice for the clinical limitations of these naturally occurring compounds is not economically feasible. These challenges demanded continuous devotions towards improving the druggability of these drugs and continue to seek structure-optimization strategies. The discovery of renewable sources including microbial origin for podophyllotoxin is another possible approach. This review focuses on the exigency of innovation and research required in the global R&D and pharmaceutical industry for podophyllotoxin and related compounds based on recent scientific findings and market predictions.

## 1. Introduction

Podophyllotoxin is an aryltetralin-type lignan isolated from species of *Podophyllum* [1,2]. Two most common sources are the rhizomes of *Podophyllum peltatum* (American mayapple) and *Sinopodophyllum hexandrum Royle* (Barberry family) [1,3]. These perennial herbs are distributed widely across the Himalayan region and Western China [4,5,6,7]. Plants with high podophyllotoxin or podophyllotoxin-analogues’ content have been extensively used in traditional medicine since a long time in different cultures [8,9,10,11]. This remarkable molecule was first isolated in 1880 by Podwyssotzki [12,13,14,15], while it had already been described as early as in 1753 by Linnaeus [16]. Due to its remarkable biological activity, podophyllotoxin has remained a subject of various investigations ever since. It is mainly obtained from the alcohol-soluble fraction of *Podophyllum* species, called podophyllin—a bitter tasting resin.

The podophyllotoxin extract has been documented for its use as a laxative, and as a remedy for various medical complications such as gonorrhea, tuberculosis, menstrual disorders, psoriasis, dropsy, cough, syphilis and venereal warts [8,9,10,11]. The podophyllotoxin-family is now confirmed to elicit various curative properties such as mitotoxic, neurotoxic, insecticidal, antimicrobial, anti-inflammatory, antispasmogenic, hypolipidemic, immunosuppressive, antioxidative, analgesic and cathartic activities [17]. Initial studies reporting its pronounced activity against cancer cells established greater interest in its antimitotic efficacy. The clinical practicality of podophyllotoxin was, however, largely tampered because of its undesirable secondary effects such as gastrointestinal toxicity, neurotoxicity, hair-loss, and bone marrow suppression, etc., which led to the finding of less-toxic derivatives or analogues. These molecules have since become a structural base for the development of new therapeutic agents.

**Figure 1:**
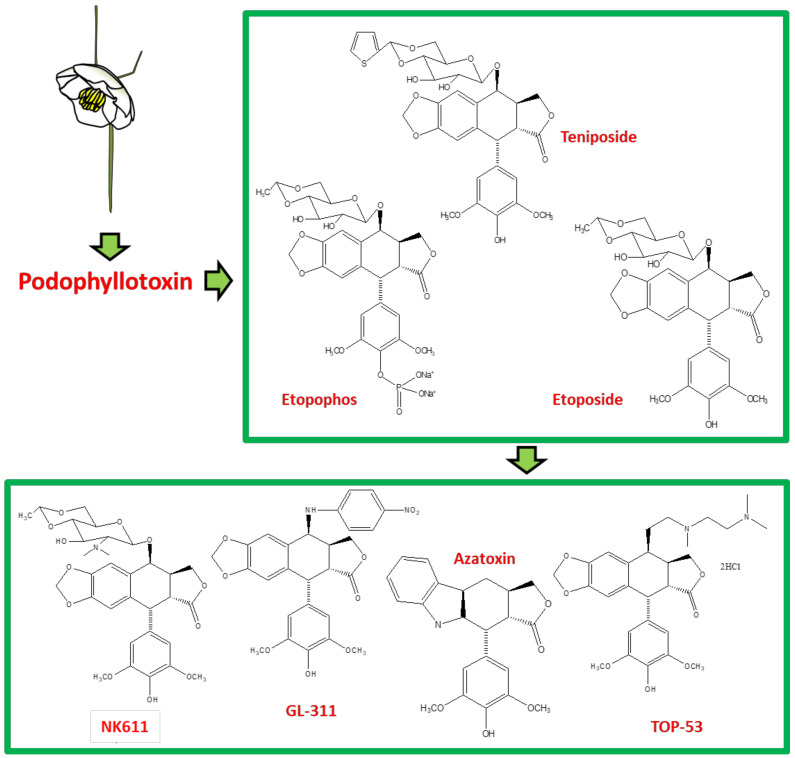
The sequential discovery of podophyllotoxin-group of drugs. FDA approved anticancer drugs etoposide, teniposide and etopophos were derived from the parent compound podophyllotoxin, which was originally extracted from mayapple plant as a curative for various diseases. As the side-effects for podophyllotoxin and its primary derivatives became evident, less toxic derivatives such as TOP-53, NK611, GL-331, azatoxin and various others were designed and synthesized.

Podophyllotoxin (C_22_H_22_O_8_) is a selective cyclolignan whose structure was first elucidated in 1930s. Extensive structural modifications of podophyllotoxin have culminated to eventually discover clinically viable anticancer drugs, namely; etiposide, teniposide [18,19,20,21,22,23,24,25] and etopophos [26], but it was not until twenty years after the discovery of these viable derivatives that their mechanism of action was understood. Podophyllotoxins were recognized to interact with the DNA and its replication process to carry out their antimitotic effects. Etiposide, for instance, inhibits DNA topoisomerase II (dnaTII) and causes cell cycle arrest in the S-phase. Podophyllotoxin is also known to prevent cell growth through inhibiting the polymerization of tubulin and thus subdue the configuration of mitotic spindles [26]. The therapeutic use of these drugs is however, in fact, hindered by myelosuppression, development of drug-resistance and their cytotoxic activity towards normal body cells. On these accounts, the synthesis of new potent podophyllotoxin-derivatives such as NK-611 [27], NPF [28], GL-311 [29], TOP-53 [30], F14512 [26], azatoxin, etc., is a continuing concern—structures of some of these derivatives are shown in Figure 1. These have thence opened a window for virtual designing of unlimited podophyllotoxin-derivatives geared at improving clinical efficacy. These studies necessitate reviewing all the recent advancement made on podophyllotoxin. Furthermore, the extinction of podophyllotoxin plant sources shifted the focus towards source alternatives, which includes the manipulation of microbes for the purpose. This review is aimed at providing an insight to podophyllotoxin research and hence helps delineate further investigations, which are needed in this field.

## 2. Structural Characteristics of Podophyllotoxin

**Figure 2:**
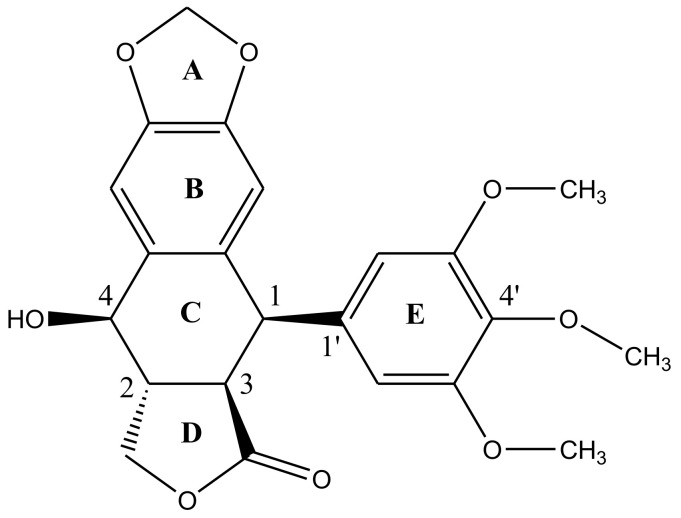
Structural representation of podophyllotoxin. The structure is constituted of 4 aromatic rings A, B, C and D arranged in an almost planar system whereas ring E is attached quasi-axially at C4 of ring C.

Podophyllotoxin has a five-ring system (ABCDE) having four chiral centers (C1-C4) in a row—Figure 2. There are five important structural characteristics of most podophyllotoxin species: (1) a tetracyclic group going from dioxole ring (A) to the lactone ring (D); (2) four oxygen atoms located at the functional groups lactone, methoxys, secondary alcohol and oxoles; (3) ring E with alpha-configuration located at position 1; (4) four asymmetrical adjacent centers and (5) the unique stereochemical properties of C4 define the molecules’ mechanism of action [31,32]. Studies have revealed, rational modification at C4 can improve the molecule’s topoisomerase II inhibitory activity, drug resistance profile, water solubility and antimitotic activities [33]. The lactone ring is known to exhibit transconfiguration, which easily epimerizes in low basic conditions [32].

## 3. Derivatives, Analogues and Hybrids of Podophyllotoxin

**Figure 3:**
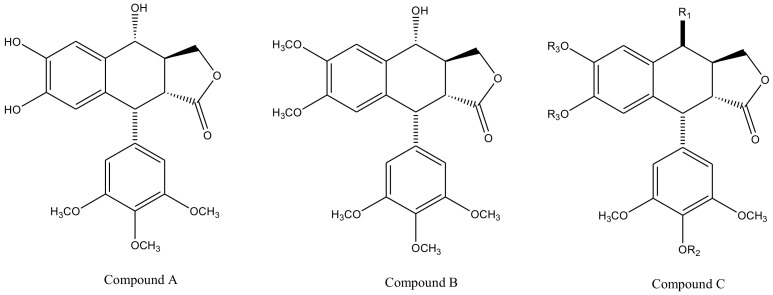

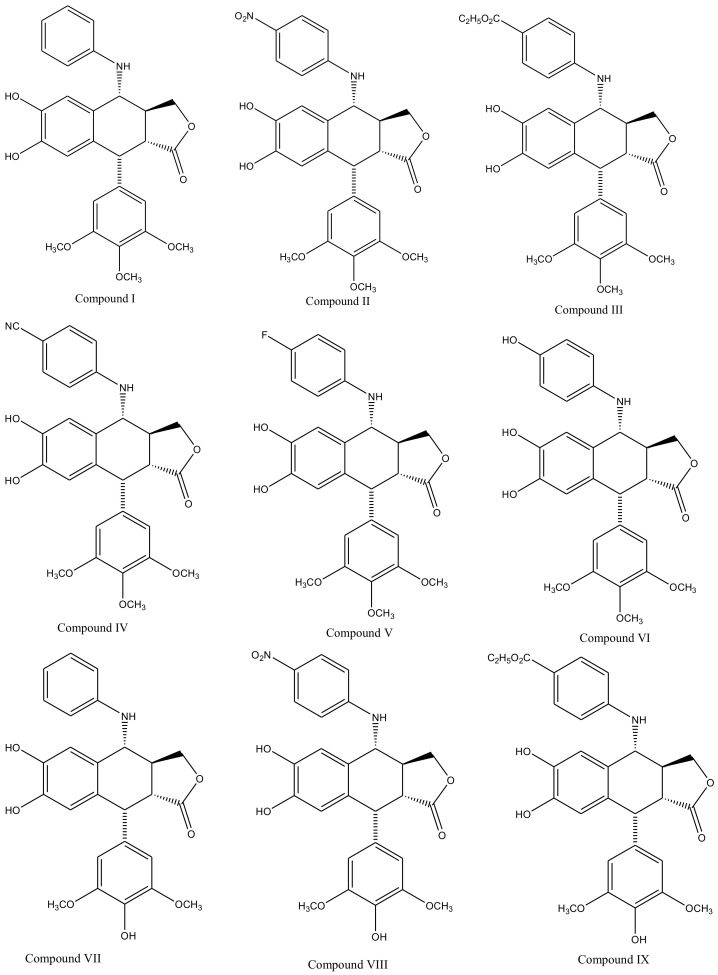

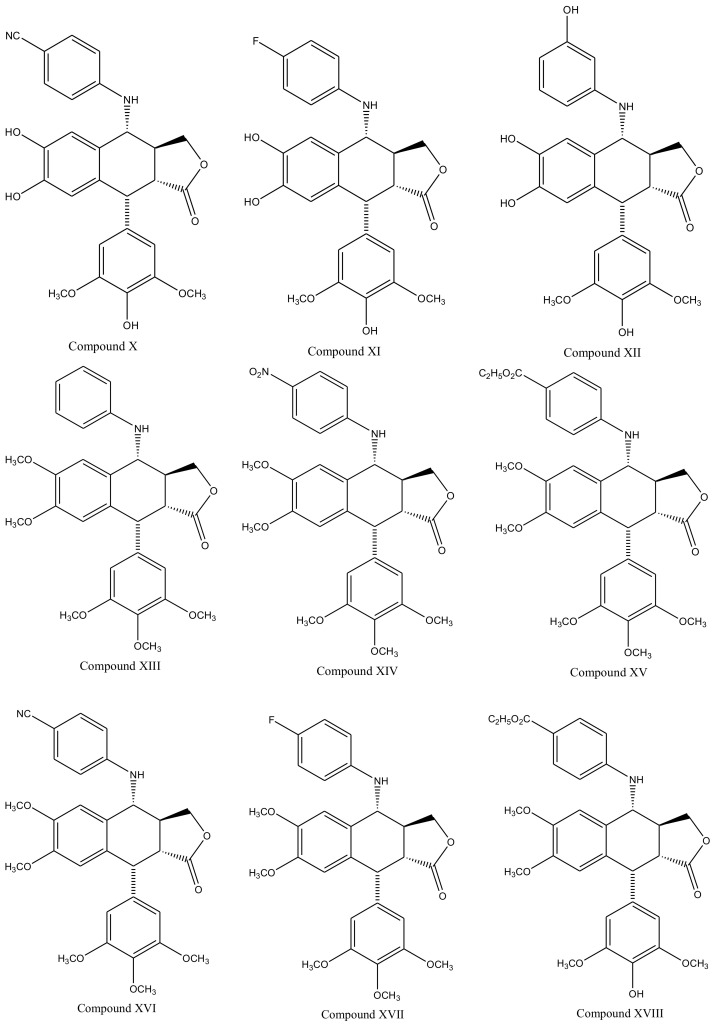

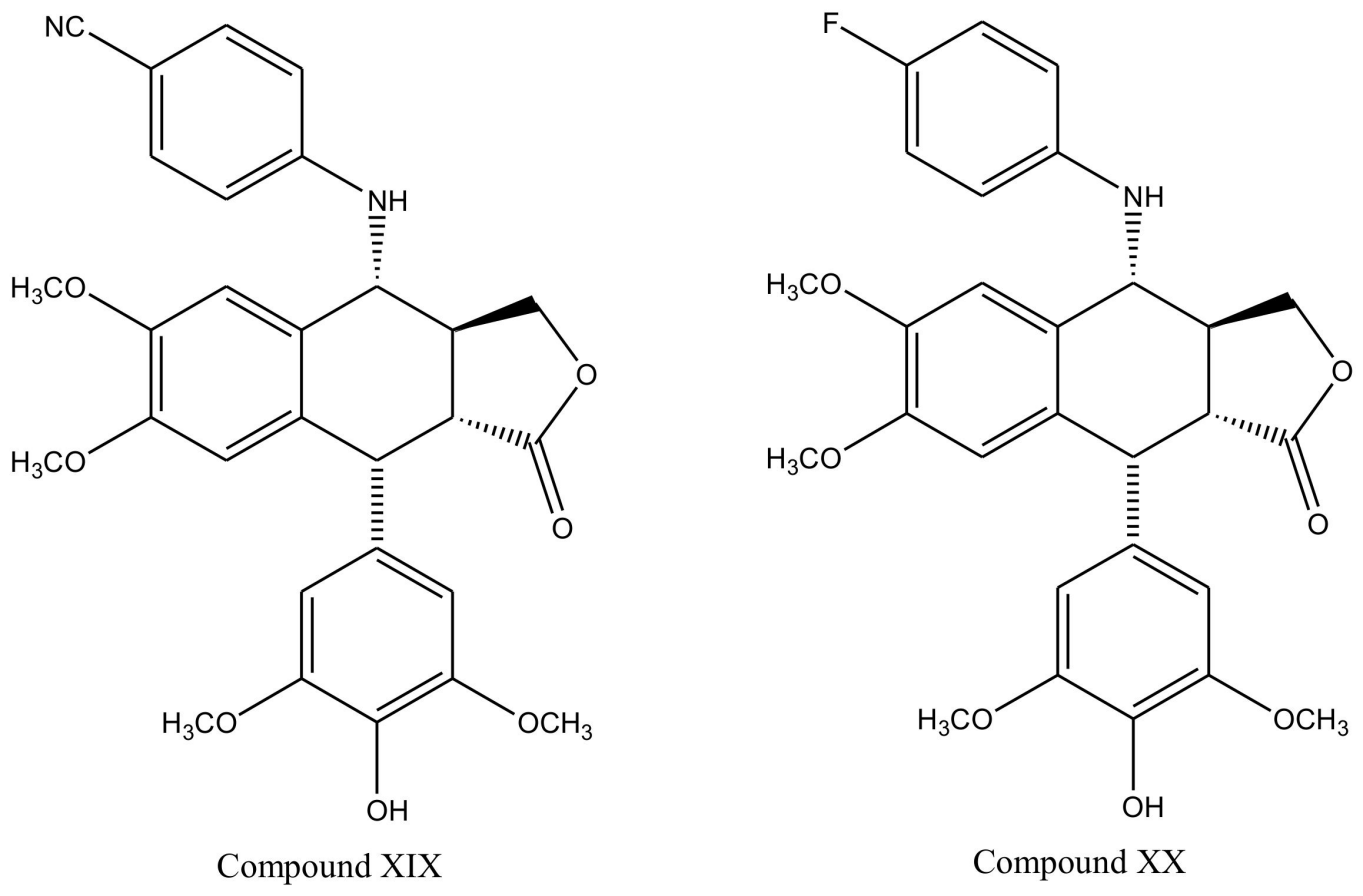
Podophyllotoxin derivatives produced by selective cleavage of ring A through Schreier’s method and the modified-Schreier method. These ring-A-open compounds, however, appeared biologically less active than podophyllotoxin itself.

Derivatives of this multifaceted molecule are synthesized as properties of its four rings. Ring A, for instance is not essential for the exhibition of antimitotic activity, and aromatization of ring C may cause displacement of axially positioned ring E—leading to a loss of cytotoxic property. Structural diversity hence brings many attractive medicinal properties and a comprehensive insight to the molecule’s mechanism of action. These derivatives were first introduced by Schreier through selective cleavage of ring A to produce a 6, 7-dihydroxy derivative (compound A), which was further methylated with diazomethane to produce yet another derivative (compound B) shown in Figure 3 [34]. Schreier’s methods were later on slightly modified to produce a large group of podophyllotoxin derivativescompound I–XXfrom compound C, Figure 3. However, the structural modifications are not alone to contribute towards the novelty of the derivatives, instead, changes in the chemical skeleton of podophyllotoxin cyclo-lignan add particularity to each individual derivative.

**Figure 4:**
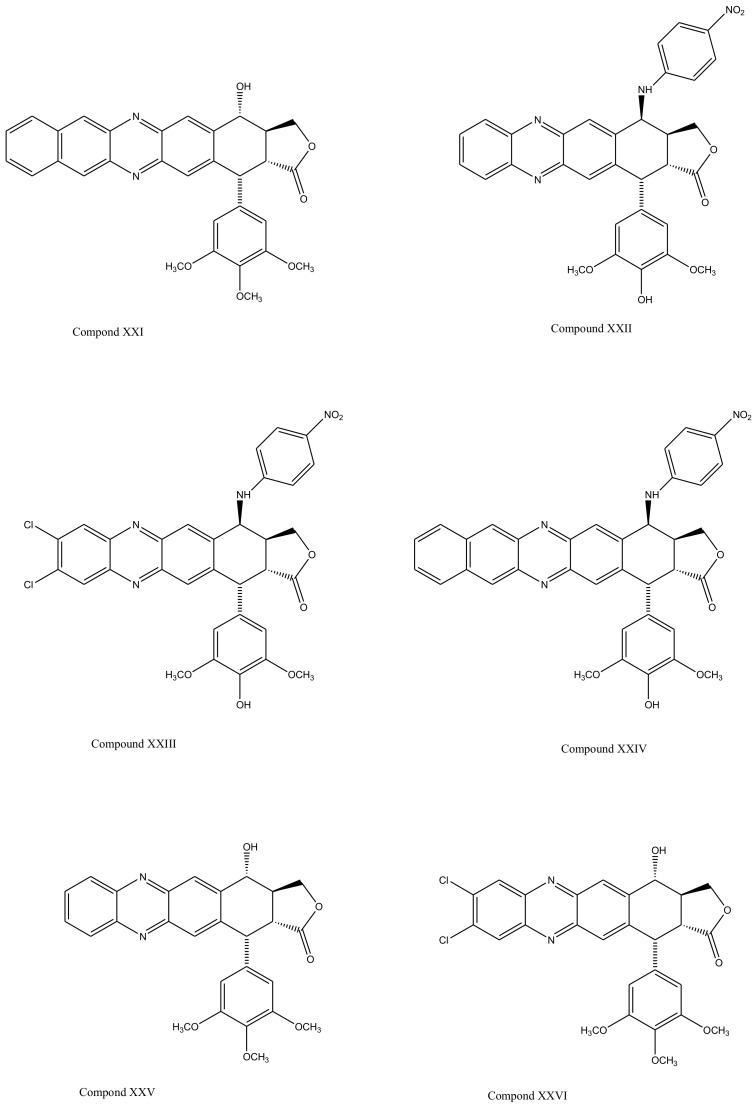
The significance of ring A for biological functionality of podophyllotoxin and its derivatives introduced three crucially significant domains in the parent pharmacophore model. Modifying these domains provided a number of new derivatives as shown (Compound XX1–XXVI). These compounds show distinct mechanism of actions in comparison to the previously discovered podophyllotoxin derivatives.

**Figure 5:**
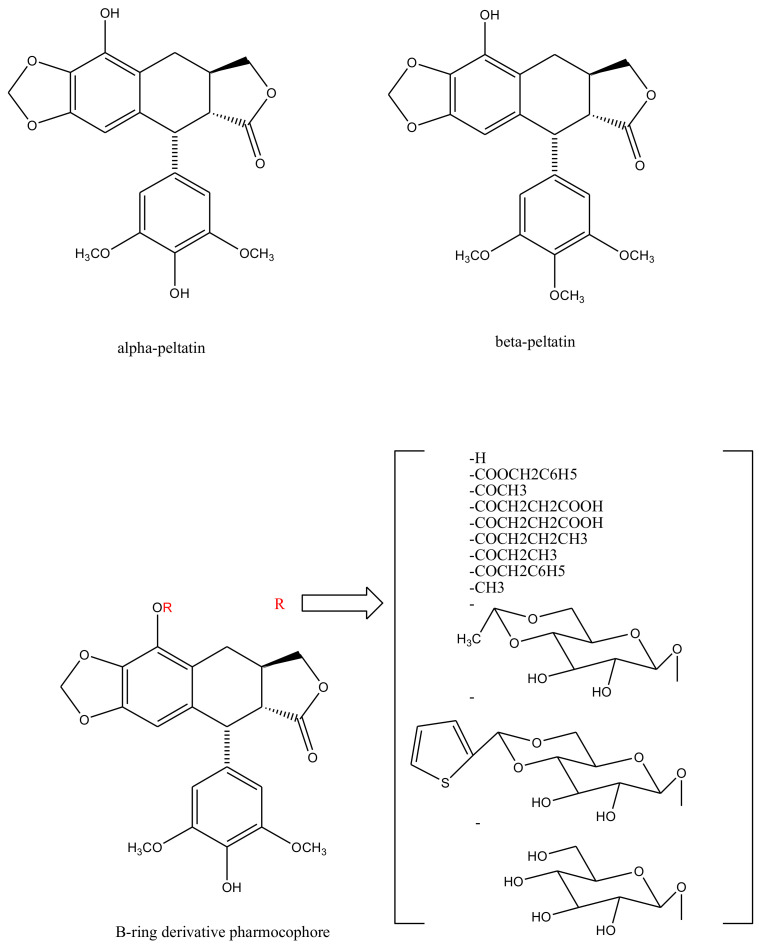
Structures of alpha-peltatin and beta-peltatin. These two B-ring modified podophyllotoxin derivatives are known to exhibit significant antiviral and antitumor activities. The B-ring modified pharmacophore was identified from these compounds and was taken further to synthesize more derivatives—as shown bracketed—none of which elicited any clinical efficacy.

With slight modifications in the podophyllotoxin skeleton, the ring-A-open compounds appeared biologically less active than podophyllotoxin itself [35]; functional studies indicate that an intact A-ring system is important for the compounds’ DNA topoisomerase II (dtopII) inhibiting activity. Based on these findings, three crucially significant domains in the parent pharmacophore model were reported [36]. Modifications at these domains further revealed the unexplored potential of novel clinically applicable derivatives (shown in Figure 4)with a mechanism of action distinct to that of the previously well-established derivative; etiposide [37]. The paucity of studies on B-ring modifications in podophyllotoxin led the investigation of α-peltatin and β-peltatin. These two compounds (Figure 5) exhibited significant antiviral and antitumor activities. In order to investigate the influence of B-ring substitutions and modifications, a series of ester- and ether-derivatives of both structures were prepared by various researchers but were reported to be biologically less active than their parent compounds [38,39,40,41,42,43].

Several C-ring modified podophyllotoxin analogues have also been subjected to extensive research. Unlike the two natural C-aromatized lignans; justicidin A and diphyllin; the many synthetic C-ring aromatized compounds were prepared but reported to have no cytotoxic activities. This observation proposed that the axial E-ring conformation was lost during synthetic preparations. The loss of the axial E-ring conformation is now known to be directly linked to the loss of molecule’s cytotoxic and dtopII inhibiting activity [44]. Notably, however, delactonized analogues of podophyllotoxin—apopicropodophyllotoxin; and its beta-isomer—β-apopicropodophyllotoxin give strong antimitotic activities, otherwise indicating the untrammelled antimitotic potential of podophyllotoxin-like compounds in the absence of the lactone ring [45,46,47]. The antitumor activity of podophyllotoxin and its derivatives is mainly due to the ability to inhibit tubulin polymerization [48], which is a property of the axially placed E-ring. The *cis*-fused lactone rings, however, modulating its selectivity from tubulin to IGF-1 (insulin- like growth factor I) receptor to trigger cell death [49,50,51].

**Figure 6:**
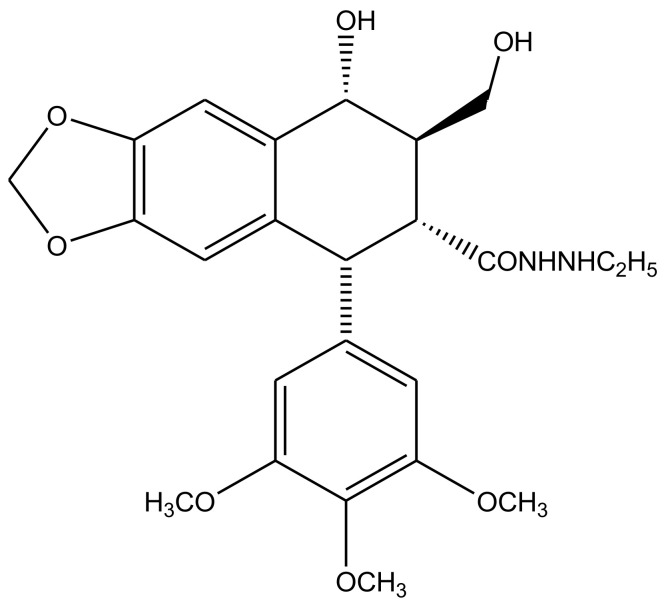
A series of delactonized D-ring derivatives have remained the focus of many studies. An ethyl hydrazide derivative, drawn here, was reportedly the only D-ring modified compound, which showed clinical efficacy, but was discontinued after occurrence of various cases of severe side-effects related to its use.

Transfused γ-lactone D ring is also strict requirement for podophyllotoxin’s antitumor activity. Since it is susceptible to isomerization, the opening of this lactone ring is undesirable for it limits the physiological lifetime of these compounds and their biological effectiveness as well. A series of delactonized D-ring derivatives were prepared [52,53,54,55,56,57] and studied by various researchers, most of which were found to be less cytotoxic than the parent compound itself. However, an ethyl hydrazide derivative (Figure 6) was found to retain its clinical efficacy but was discontinued due to severe adverse aftereffects [58]. Studies that later discovered immunosuppressive ability of these derivatives were attempted to revive D-ring modified podophyllotoxin—antitumor drug preparations [59,60]. 4′-d-β-DMEP (4′-d-β-Demethylepipodophyllotoxin) is a natural lignin, which is used in chemotherapy as an aglycon of etoposide [61,62]. 4′-d-α-DMEP is a novel biological derivative of 4′-d-β-DMEP reported by Jia et al. (2020), which has a higher antitumor activity [63].

**Figure 7:**
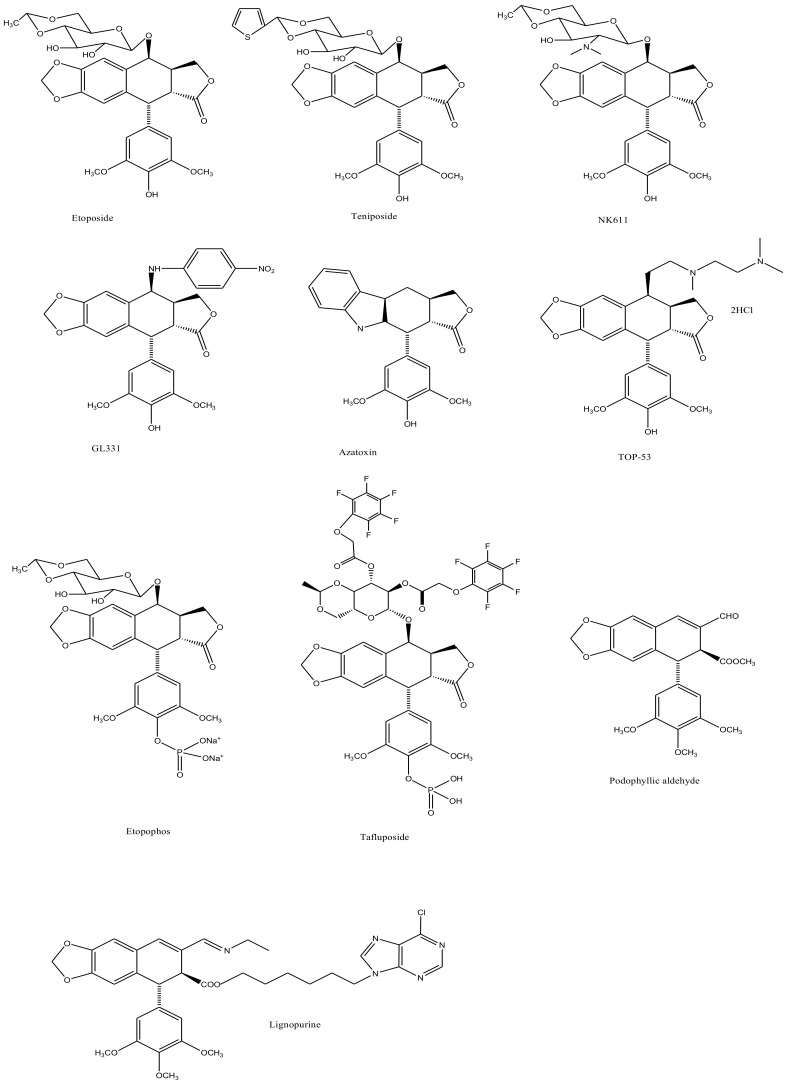
The figure shows structural illustrations of E-ring modified derivatives much of which have proved to be clinically significant.

E-ring modifications have only actually focused on enhancing E-ring’s involvement in the cytotoxic mechanism of the compound. With an increased access to the compounds’ physicochemical behavior, a variety of exciting derivatives with successful clinical application have been developed, such as those shown in Figure 7 [64,65].

## 4. Plant Sources of Podophyllotoxin

A variety of plant species belonging to approximately over 60 vascular families have been reported as viable sources of podophyllotoxin, which includes genera other than the genus *Podophyllum*, such as *Diphylleia* and *Dysosma* (beriberidaceae), *Haplophyllum* (Rutaceae), *Thuja*, *Jeffersonia*, *Anthriscus* (Apiaceae), *Callitris* and *Thujopsis* (Cupresceae), *Hernandia* (Hernandiaceae), *Nepeta* and *Thymus* (Labiaceae), *Catharanthus* (Apocynaceae), *Teucrium*, *Hyptis* (Verbenaceae), *Commiphora* (Burseraceae), *Polygala* (Polygalaceae), *Linun* (Linaceae), *Juniperus* and *Cassia* (Fabaceae) [17,26]. Podophyllotoxin is found accumulated mostly in the roots and rhizomes of these plants but have also been detected in their stems, roots, seeds, fruits, leaves, woody parts and, in some cases, from the associated endophytic microorganisms. However, *Podophyllum hexandrum* Royle has been reported to contain higher amounts of podophyllotoxin (4.3%) compared to other podophyllotoxin producing plant species [66]. The various podophyllotoxin producing plant species have been tabulated below (Table 1).

## 5. Biosynthesis of Plant Derived Podophyllotoxin

**Figure 8:**
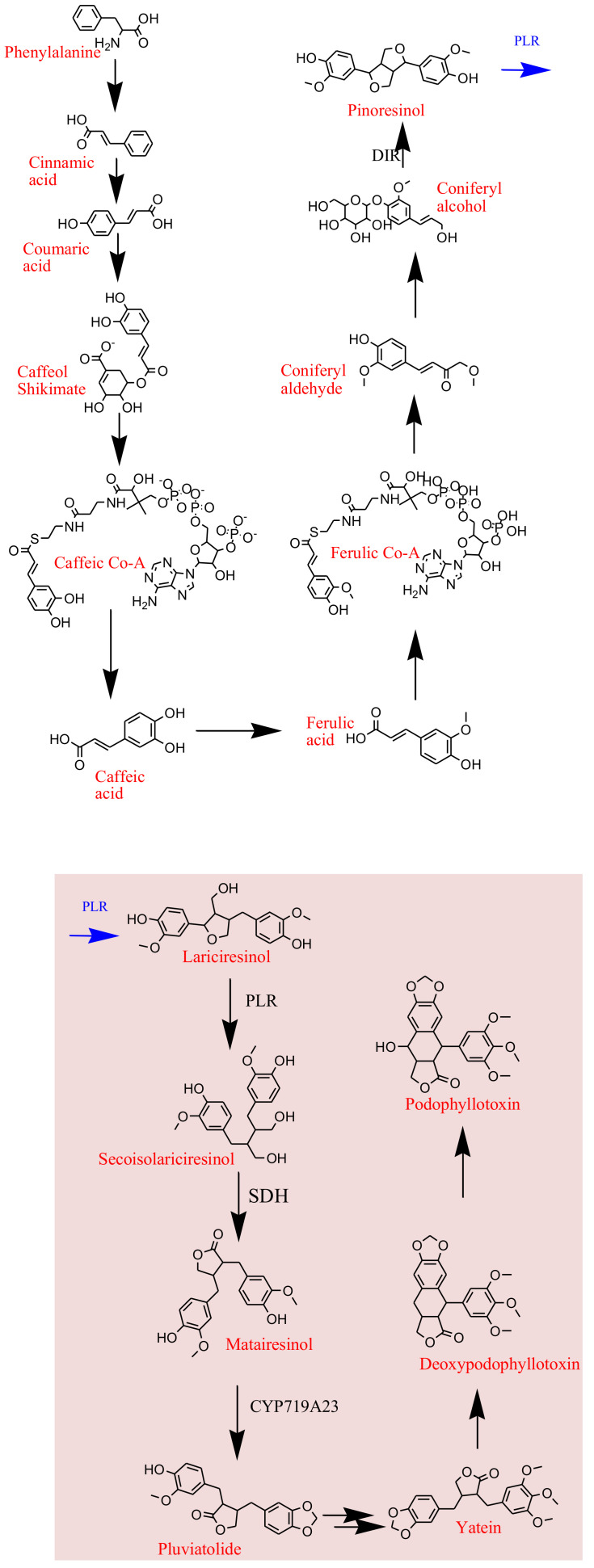
The production of podophyllotoxin in plants takes place in 33 steps where coniferyl alcohol acts as the precursor. The pathway is called phenylpropanoid pathway. In the early steps of podophyllotoxin biosynthesis coniferyl alcohol in formed in nine steps from phenylalanine. Coniferyl alcohol then undergoes a site-selective and unusual enantio-dimerization to form (+)-pinoresinol. Pinoresinol is then reduced to (−)-secoisolariciresinol, which is catalyzed by a dehydrogenase to (−)-matairesinol. The next intermediate formed is (−)-pluviatolide, which is methylated to (−)-5′-desmethoxy-yatein that is converted to the yatein. Yatein is the intermediate that gets converted to the end product; podophyllotoxin [109].

The understanding of plant pathways is comparatively absolute to the microbial pathways characterized to date. Plant genes that are involved the biosynthetic pathways of various plant-derived clinical drugs remain obscure, hence preventing the transfer of which to heterologous hosts for industrial production. The complete biosynthetic pathway (Figure 8) for podophyllotoxin was elucidated only recently in 2015, allowing more facile access to its natural and processed, clinically significant derivatives—otherwise difficult to synthesize on an industrial scale [108].

## 6. Microbial Sources of Podophyllotoxin

Biotechnological procedures for podophyllotoxin production were introduced as a means to overcome its large scale production challenges, which includes the extinction of various podophyllotoxin-plant sources, slow growth of plant sources, exacting constructs and low yields. Micro-organisms, however, demonstrate an enormity of biodiversity, which overdo those of plants. Fungal sources, for instance, have rendered to be a much fruitful alternative to somatic embryogenesis, tissue culturing or macropropagation techniques. The main fungal sources used for industrial podophyllotoxin production are; *Trametes hirsuta* [110], *Fusarium oxysporum* [111], Fungus *Alternaria* [112], *Mucor fragilis* [113], *Fusarium solani* [114], *Trametes histuria* [110], *Sebacina vermifera*, *Phialocephala fortinii* [115] and *Aspergillus fumigatus* [101,116]. The endophytic sources of podophyllotoxin are also tabulated below Table 2 [111] describes the process of podophyllotoxin production from *Fusarium oxysporum,* which lives on *Juniperus recurva*. The outcome suggests *F. oxysporum* to be a promising contender for industrial podophyllotoxin production. In 2009, the specie *Juniperus communis* L. Horstmann was also found to produce deoxypodophyllotoxin, which shared remarkable structural relativity to the lignan of podophyllotoxin [117]. No bacterial sources of podophyllotoxin have yet been identified.

## 7. Parametric Analysis of Podophyllotoxin Biosynthesis

Studies report five major parameters, which influence the biosynthesis of podophyllotoxin; these include luminosity, chilling units/hours, macro- and micro-nutrients and soil’s pH and nutrient availability. Any fluctuation in the strength of which can result in altered podophyllotoxin yields. In the presence of red-light, for instance, increased the overall product formation in comparison to light of other wavelengths [123]; likewise, chilling units set at 4 °C were reported to have caused a 5-folds increase in product yield. The variation in concentrations of macro- and micro-nutrients such as glucose, nitrogen, NO_3_^−^, PO_4_^3−^, Na^+^, Fe^2+^, Mn^2+^, etc., has shown similar correlation with podophyllotoxin production. Moreover, the acidity or alkalinity levels of the soil in cases for podophyllotoxin producing plants also demonstrate yield modifications. These parameters are tabulated and discussed below Table 3.

## 8. Pharmacological Significance of Podophyllotoxin and Its Derivatives

Podophyllotoxin and its derivatives possess a wide-spectrum of pharmacological potential. The achievability of broad chemical modifications brings the podophyllotoxin pharmacophore its diverse applicability as a medicinal compound—tabulated in Table 4. Antineoplastic activity is the most outstanding of all its clinical properties. Various studies validate podophyllotoxin derivatives including etopophos, teniposide, etoposide, etoposide phosphate, GL331, NK-611, TOP53 and NPF as anticancer drugs [64,128,129,130,131,132]. Many clinical studies have reported the efficacy of these compounds against various forms of cancer including lung cancer, Wilmstumours, diverse types of genital tumors such as carcinoma verrucosus and for non-Hodgkin lymphoma, multiform glioblastoma lymphoma and nonlymphocytic leukemia [17]. Podophyllotoxin has also shown activity against various (multi) drug resistant tumor cells. As an example, Hu and coworkers presented a 4-β-anilino-podophyllotoxin derivative as a potential anticancer drug against the KB/VCR cells in both conditions; in vivo and in vitro [133]. For the development of new anticancer drugs, podophyllotoxin containing analogues are of prime focus in recent studies. Ming et al. [134] explained that an endophytic fungus named as *Dysosma versipellis* has both anticancer and antimicrobial properties. The aqueous extract of *Podophyllum peltatum* [41] fractionated by reverse-phase chromatography was observingly the most active component that was found to cause the inhibition in the replication process of herpes simplex type 1 virus and measles.

Moreover, picropodophyllotoxin, deoxypodophyllotoxin and peltatins are also determined as useful antiviral compounds [135,136,137]. Activity against Sindbis and cytomegalovirus has also been recorded [138]. They either decrease the capacity of the infected cell to release virus or restrain these viruses in the replication cycle at an essential early stage, following the virus absorption into cells. Not only this, podophyllotoxin can also be used for treating *Condyloma acuminatum* that is usually caused by HPV (papilloma virus) [139] and for treating other perianal and venereal warts [140]. With a goal to achieve better therapeutic effectiveness, cocktail therapies are currently in use with other registered chemotherapeutic agents, combined with additional techniques that are beneficial in fighting against cancer and other viral infections. Podophyllotoxins with interferon therapy has shown greater effectivity against genital human infections, combination with cisplatin, on the other hand, is useful for treating neuroblastomas. Recently a study reports the study progression of etoposide in phase II clinical trials (ClinicalTrials NTC04356690) as a redeveloped drug to treat COVID-19 patients suffering with cytokine storm complication [141]. Similarly, in recent years podophyllotoxin derivatives have shown some interesting insecticidal activities against the larvae of *Brontispa longissima* and *Mythimna separata* [142]. Furthermore, derivatives are also being synthesized and used for the treatment of malaria and psoriasis [143]. Podophyllotoxins also hold dermatological significance and prove to be potential therapeutic agents for psoriasis vulgaris. Podophyllotoxin exhibited considerable ichthyotoxic activity and phyto growth inhibitory activities, even though the effects were weaker as compared to deoxy podophyllotoxin, in all cases observed [144,145].

Besides the major antiviral and anticancer activities, podophyllotoxin and its derived compounds have also exhibited some further interesting activities such as anti-trypanosomal activity, antimelanocortin-4 receptor (MC4R) activity, antioxidative activity, immunosuppressive, antioxidative, antispasmogenic, hypolipidemic, emetic, laxative and anti-inflammatory activity. Moreover, during the last century, many trials were carried out in an attempt to cure diseases such as syphilis, cough, gonorrhea, dropsy, gout, psoriasis, tuberculosis, tumor, menstrual disorders and venereal warts by using podophyllotoxins. According to Hartwell et al. (1951), podophyllotoxin is particularly abundant in the genus *Podophyllum* that has been utilized since ancient times as bothanthelminthic and cathartic agents, for medicinal purposes [3]. Podophyllotoxin is demonstrated as a promising and relatively safe drug, for treating genital warts and is also an active ingredient of registered wartec ointments and condylox liquids.

**Table 4:** Pharmacological activities of podophyllotoxin and its derivatives.

Activity	Podophyllotoxin Derivative	Mechanism	Conclusion	Ref.
Cytotoxic activity	Cleistantoxin	Activity was checked against MCF-7, MCF-7R, KBand HT29 cancer cell lines	Cleistantoxin showed strong cytotoxic activity	[146]
Antibacterial activity	New precursors of podophyllotoxin were synthesized, and screened to check their antibacterial activity	Activity was checked against *Klebsiella pneumoniae, Streptococcus faecalis*, *Citrobacter* sp., *Pseudomonas aeruginosa*, *Escherchia coli, Salmonella typhi* and *Shigella sonnei*	Ethyl-2-(3′-methyl-4′-methoxybenzoyl)-3-(4″ methoxyphenol)-cyclopropane-1-carboxylic acid and Ethyl-2-(3′-methyl-4′-methoxybenzyol-3-1 3″, 4″-dimethoxyphenyl)-cyclopropane-carboxylic acid, both of them showed significant antibacterial activity	[147]
Antitumour activity	VP 16-213 (NSC-141540)	Activity was checked against L1210 ascites tumour in N/D mice	In 24 hours, divided treatment after every 3 hours, resulted in significant cure, hence, VP 16-213 is a cell cycle specific drug	[148]
Insecticidal activity	20 podophyllotoxin analogues were tested	Activity was checked against fifth-instar larvae of *Brontispa longissima* in vivo	Among 20 analogues Deoxypodophyllotoxin showed more protential for insecticidal activity than a commercial insecticide (toosendanin)	[149]
Antineoplastic activity	New hybrids of podophyllotoxin and indirubin	Activity was checked against human leukemia cancer cells as a multifunctional anti-MDR agent	Podophyllotoxin-indirubin hybrid (Da-1) showed potential to overcome drug resistance. It is a novel hybrid havingpotent antiproliferative activity	[150]
	Cyclolignans, derived from podophyllotoxin	Activity was checked againstA-549 human lung carcinoma, P-388 murine leukemia and HT-29 colon carcinoma	A number of substances were active in assay at concentrations below 1 pM; deoxypodophyllotoxin being the most potent compound in all cases	[151]

## 9. Patents

In the past decade, numerous attempts have been globally to produce derivatives or compositions of podophyllotoxins in order to overcome their natural dose-limiting toxicity, poor biodistribution and low aqueous solubility. These inventions have found extensive application for effective chemotherapeutic purposes. These inventions have succeeded at demonstrating sufficient activity and can considerably contribute to future studies, some of these new derivatives are tabulated below (Table 5).

**Figure 9:**
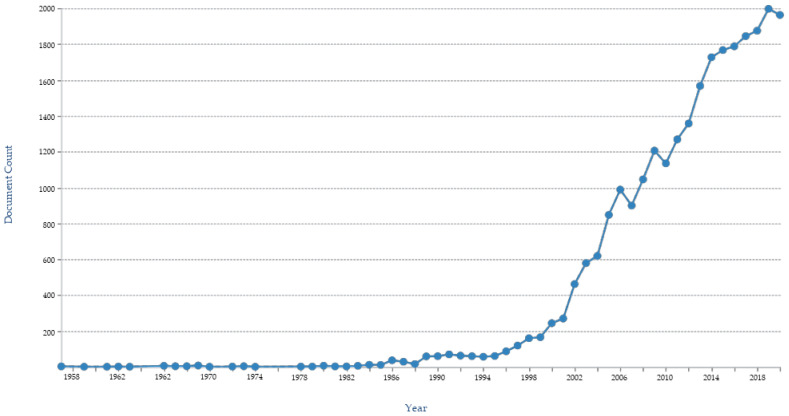
Year wise patent publications filed on podophyllotoxin.

**Figure 10:**
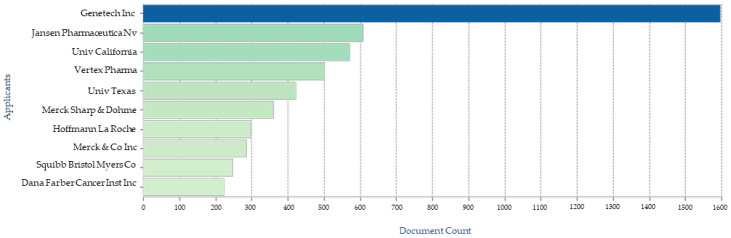
Key patent filing companies on podophyllotoxin.

**Figure 11:**
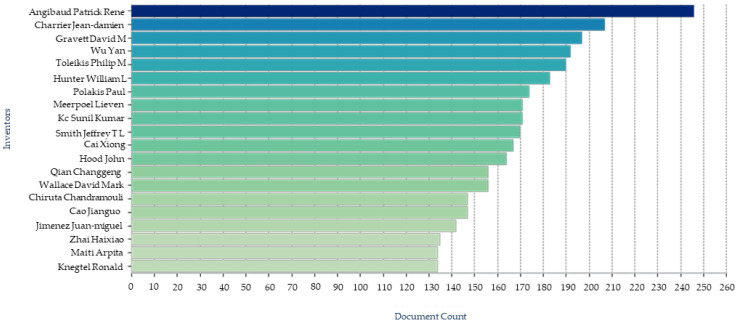
Key inventors in the podophyllotoxin field.

**Figure 12:**
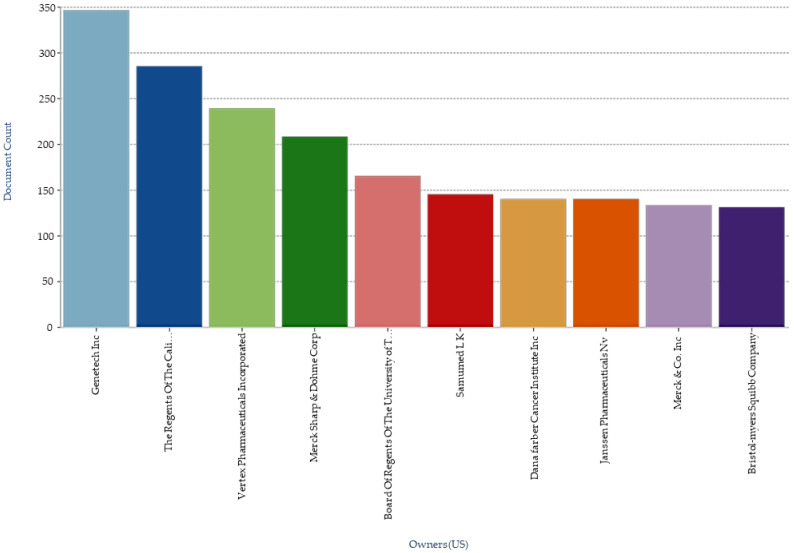
Top US owners of patents related to podophyllotoxin.

The great interest of institutional researchers and pharmaceutical companies is also validated by a surge in number of patents protecting various inventions involving podophyllotoxin. Searching the term “podophyllotoxin” gave 93,446 results in the Google patent database while 26,582 in the Patent Lens database with the first patent application filed in 1967-12-06 and granted in 1970-08-18. When “podophyllotoxin” is used as keywords for the the patents and/or patent applications annual filed (Figure 9), key applicant companies (Figure 10), key inventors (Figure 11), and key owners based in US (Figure 12) [160,161]

## 10. Conclusions

Novel approaches towards structure optimization and the finding of alternative sources can help unravel less toxic, more efficient and easy to yield podophyllotoxin and its derivatives. Being known for decades as the main bioactive compound in various traditional medicinal formulas, these compounds were later approved for clinical use against a number of health conditions. In the recent pandemic, various known drugs were repurposed against the novel coronavirus strain 2019. Amongst this struggle of trial and error, etoposide—a podophyllotoxin derivative well cited for its antineoplastic activity—has also been redeveloped to manage cytokine storm complication in COVID-19 patients. This finding opens new potential for podophyllotoxin, its derivatives and its congeners to be explored. For as the growing demand for podophyllotoxin is increasing, plant sources are proving to be an unreliable option for the future, this explains the exigency of utilizing microbial biotransformation as a considerable approach along with the need of devoted effort towards rationally designing the new generation of the group of podophyllotoxin-derived compounds.

## Figures and Tables

**Table 1:** Various plants from different plant families, which are well reported as sources of podophyllotoxin.

S. No.	Family; Plant(s)	Part Used	Derivative	Ref.
1	**Apiaceae;** *Chaerophyllum aurium*	Extract of subaerial part	Deoxy podorhizone and deoxypodophyllotoxin	[67]
2	**Acanthaceae;** *Justicia heterocarpa*	Extract of aerial part	Podophyllotoxin lignan	[68]
3	**Berberidaceae;** *Jeffersonia diphylla,* *Dysosma pleiantha,* *Dysosma versipellis* var. *tomentosa*, *Dysosma versipellis*	Extract of root, Culture of Callus,	Podophyllotoxin, kaempferol and quercetin, Podophyllotoxin derivatives, 4-demethylisopodophyllotoxin	[69,70,71,72,73]
4	**Burseraceae;** *C. incisa* *B. permollis* *B. fagaroides* *B. microphylla* *B. konkinensis*	Resin, Stem bark, Exudate, Stem, Extract of root	Derivatives of podophyllotoxin, Deoxypodophyllotoxin, 4-demethyldeoxypodophyllotoxin	[74,75,76,77,78]
5	**Broginaceae;** *L. erythrorhizon*	Extract of needle leaf	Podophyllotoxin and derivatives	[79]
6	**Cupressaceae;** *J. sabina* *J. virginia* *J. conferta* *C. preissii* *J. depressa* *C. rhomboidea* *J. virginiana* *J. chinensis* *J. horizontalis* *J. davurica* *C. endlicher* *J. thurifera* *C. drummondii* *C. intratropica* *J. squamata* *J. chinensis* *C. collumelaris* *J. scopulorum* *J. lucayana* *J. virginiana* *J. silicicola* *J. viriginia* *T. occidentalis*	Extract of needle leaf, Culture of suspension, culture of callus, needle leaf aqueous suspension, extract of needle leaf, Extract of stem, Extract of wood, Aerial part, Twig and extract of needle leaf	Podophyllotoxin and derivatives, Deoxypodophyllotoxin, 5-methoxypodophyllotoxin	[79,80,81,82,83,84,85,86,87,88,89,90,91,92]
7	**Linaceae;** *L. perenne* *L. scabrellum* *L. thracicum* spp. *L. capatitum* *L. elegans* *L. austriacum* *L. arboreum* *L. hirsutum* *L. usitatissimum* *L. scabrellum* *L. strictum* spp. *L. album* *L. flavum* *L. mucronatum* spp. *L. persicum* *L. nodiflorum* *L. mucronatum*	Culture of hairy root, Culture of suspension, Seed extract, root extract, aerial part, Callus, Tissue culture, Aerial Tissue culture, cell culture	Podophyllotoxin, Podophyllotoxin derivatives, 5-methoxypodophyllotoxin, 6-methylpodophyllotoxin, 6-methoxypodophyllotoxin	[86,91,93,94,95,96,97,98,99,100,101,102,103,104,105,106,107]

**Table 2:** Various fungal sources reported as sources of podophyllotoxin.

Class	Fungal Endophyte	Host Plant	Ref.
Sordariomycetes	*Fusarium oxysporum* *Fusarium solani* *Fusarium* sp. *Pseudallescheria* sp.	*Juniperus recurva, Podophyllum hexandrum, Dysosma versipellis, Sinopodophyllum hexandrum*	[111,114,118,119]
Agaricomycetes	*Trametes hirsuta*	*Podophyllum peltatum*	[110]
Leotiomycetes	*Phialocephala fortinii*	*Podophyllum peltatum*	[115]
Dothideomycetes	*Alternaria tenuissima*	*Sinopodophyllum emodi*	[72]
Eurotiomycetes	*Aspergillus fumigatus, Penicillium implicatum*	*Juniperous communis, Dysoma veitchii, Diphylleia sinensis*	[117,120,121]
Ascomycetes	*Monilia* sp.	*Dysoma veitchi*	[122]
Zygomycetes	*Mucor fragilis*	*Sinopodophyllum hexandrum*	[113]

**Table 3:** The influence of varying parameters on yield of plant-biosynthesized podophyllotoxin.

Parameters	General Effect	Sub-Parameter	Podophyllotoxin Yield	Ref.
Light	Light can increase or decrease the biosynthesis of podophyllotoxin.	Red light	Substantial increase	[123]
		Blue light	Slight increase	
		White light	Decrease	
Chilling Temperature	Chilling temperature can increase or decrease the biosynthesis of podophyllotoxin	4 °C	5-folds increase	[124]
		10 °C	3.33-folds increase	
Macro Nutrients	At different concentrations major nutrients can increase or decrease the biosynthesis of podophyllotoxin	Glucose concentration	Highest levels of yield at 60 g/L	[125]
		Phosphate concentration	Highest levels of yield at 1.25 mM	
		Nitrogen concentration	Highest levels of yield at 60 mM	
Micro Nutrients	Different ions can influence the yield of podophyllotoxin	NO_3_^−^, PO_4_^3−^, Na^+^, Fe^2+^, Mn^2+^	Positive correlation	[126]
		SO_4_^2−^, K^+^	Negative correlation	
		Mg^2+^, Ca^2+^, Cu, Zn	No correlation	
Soil Nutrients	Podophyllotoxin production can be increased or decreased by acidic or basic pH and nutrient availability	pH	Podophyllotoxin content was increased significantly (more than 6.62%) when pH of soil was 4.82	[127]
		Nitrogen	Podophyllotoxin content was increased significantly when nitrogen content was 2.7%	
		Carbon	Podophyllotoxin content was increased significantly when soil organic carbon content was 3.32%	

**Table 5:** Novel patented podophyllotoxin derivatives.

Sr. No	Formulae	Patent Number	Medical Application	Ref.
1	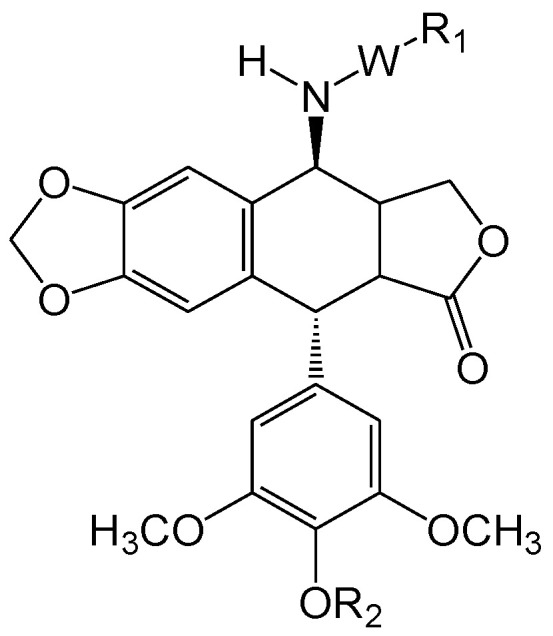	US-8158809-B2	Cancer treatment	[152]
2	-NA-	WO-02/102804-A1	Inhibit Insulin-like growth factor-1′s tyrosine-phosphorylation activity	[153]
3	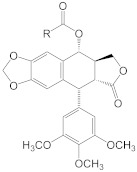 R: -CH2NHCOR2, -CH(OH)CH(Phe)(NHCOR3), & chains containing: pyrrole, thiazole, indole, naphthelene, phenyl, quinoline, pyrazine or pyridine groups.	WO-03082875-A3	Cancer treatment	[154]
4	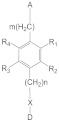 And a salt thereof.	US-10639295-B2	Elevated pharmaceutical efficacy in terms of drug concentration buildup in body	[155]
5	Assignee: Council Of Scientific and Industrial Research—status: pending	US-2020123171-A1	NA	[156]
6	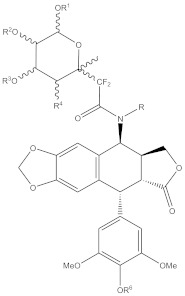	US-8236935-B2	Cancer treatment	[157]
7	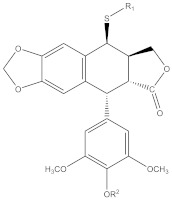 R1: substitutively selected from the following 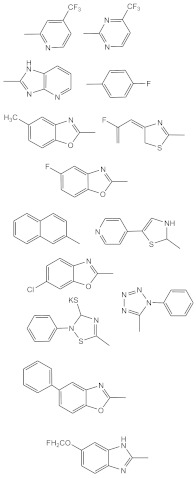 R2: -H, or –CH_3_	US-2020216462-A1	Improved antitumor activity, reduced undesired cytotoxicity	[158]
8	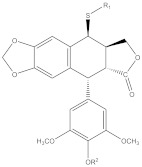 R1: Any of the following 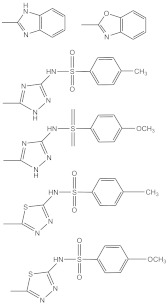 R2: -H or –CH_3_	US-9828386-B2	Elevated antitumor activity than podophyllotoxin or 4′demethylepipodophyllotoxin	[159]

## Data Availability

All the data produced here is available and can produced when required.
